# Psilocybin and Mental Health–Don't Lose Control

**DOI:** 10.3389/fpsyt.2018.00293

**Published:** 2018-07-03

**Authors:** Joseph M. Barnby, Mitul A. Mehta

**Affiliations:** Department of Neuroimaging, Institute of Psychiatry, Psychology & Neuroscience, King's College London, London, United Kingdom

**Keywords:** psychedelics, psilocybin, depression, clinical trials, methodology

Psilocybin—the hallucinogenic pro-drug in magic mushrooms—has recently dominated the popular narrative on new approaches to treating depression. For example, recent papers from John's Hopkins (1) and Imperial College (2, 3) demonstrate the potential for psilocybin to promote positive lifestyle changes, and as an intervention for treatment-resistant depression, respectively. They also provide an opportunity to highlight two recurrent issues with psychedelic research which we place in the context of more recent data in this article. Specifically, the absence of a placebo, active control, and lack of additional measures still limits the informative nature of these studies.

In this article we hope to illustrate two key issues from the recent psychedelic literature, namely (a) the use of support models (psychotherapy assisted treatment) in the absence of a placebo or active control group and, (b) the importance of placebo and active control treatment to interpret neural changes, particularly in the light of potential placebo responders. These are not new issues and indeed are acknowledged by authors of recent studies in this field (1–3), but the specific contributions of these control conditions is not straightforward.

The more recent studies mentioned complement the previous studies using psilocybin as a means to reduce anxiety and depression in terminal cancer patients, which were at the time hailed as the “most rigorous double-blind placebo-controlled trials of a psychedelic drug in the last 50 years” (4). Despite the growth of supportive studies and use of converging multimodal evidence our understanding of how psilocybin may be beneficial is in its infancy. Progress needs to be made in explicitly understanding the cognitive and neural mechanistic process by which psilocybin works, and also how specifically efficacious psychedelic treatment is outside of the sample used in these trials. This is pointed at by McCorvy et al. (5) in a commentary on the studies by Griffiths et al. (6) and Ross et al. (7), but we hope to expand on these future directions in light of new evidence.

Griffiths et al. (1) studied the added-value of using supportive therapy to instill positive lifestyle change alongside psilocybin. Aside from meditation practice, high support and standard support interventions provided comparable results in terms of increased mystic and spiritual practice ratings when participants were given two different doses of psilocybin (20 mg/70 kg and 30 mg/70 kg). In essence, variance in support did not greatly impact the response observed in high-dose sessions.

There is a paucity of evidence attempting to understand the dose-response effects of psilocybin. For this reason, these results significantly extend our understanding, but additional groups would add rigor. Inclusion of all four possible conditions—low/high psilocybin and standard/high support—in addition to psilocybin/no support and placebo standard/high support would strengthen Griffiths et al.'s (1) study. However, there are limitations. First, completing a multi-arm design is practically difficult and resource intensive. Second, guidelines from the same group (8) argue against the use of psilocybin without support. However, they do not argue against the use of placebo with support, or low dose psilocybin with high support. Lack of these groups constrict conclusions and renders the dose-response effect from both psilocybin and support groups unclear.

Carhart-Harris et al. (2) explored neurobiological changes associated with clinical response in patients with depression. All participants showed a reduction in symptoms post-treatment-this response was significantly associated with lower cerebral blood flow in the amygdala. In addition, altered connectivity differed between responders and non-responders, as defined at 5-weeks post treatment (but not when defined on the post-treatment scan day), in networks previously associated with depression. While supporting the safe use of psilocybin for treatment-resistant depression (2), the full value of the neurobiological supporting data remains limited by lack of a comparator group—the promising imaging changes cannot be attributed to psilocybin unequivocally. Nonetheless, brain imaging data alongside the initial, lasting depressive symptom reduction provides converging evidence for the effects of psilocybin - bringing mechanistic plausibility to the trial evidence. The use of a group given another drug treatment or only a course of CBT would help elucidate potential differences in neural mechanisms—differences recently theorized (9).

One particular consideration of these data is taking into account how much therapeutic value psilocybin may be having when acknowledging the natural time-course of depression. After severely depressed participants were included in the study, it is unclear to what extent symptoms may have diminished naturally without intervention. Therefore, the difference between pre and post-treatment scans may be less indicative of treatment effect. If participants were scanned very soon after inclusion, this poses more of an issue. One method of remedying this issue may be the inclusion of a control group–this is however hard to implement in practice when dealing with psychedelics.

It would be easy to simply point the finger at the use of open-label, rather than placebo-controlled designs in these limitations. But, control groups for psychedelic treatments are problematic, not least because the profound and unusual experiences associated with these compounds lead to unblinding. Of course, this does not mean that the impact of placebo should not be studied. An attempt at incorporating a placebo condition for the psychedelic ayahuasca has been posted on BioRxiv (10). Authors tested the effect of ayahuasca on depression symptomatology (*n* = 14) compared to a bespoke placebo (*n* = 15). Ayahuasca is a brew—traditionally used in ritual by indigenous populations of the Amazon Basin—with a mixed action that includes agonist activity at serotonin receptors. Authors report rapid and significant antidepressant effects from a single dose compared to a placebo. The placebo was a liquid carefully designed to simulate the sensations of ayahuasca, such as taste, color and gastrointestinal distress. Additional procedures were used to maintain blinding, and it is noteworthy that five patients misclassified placebo as ayahuasca.

The incorporation of neuroimaging data would still benefit from “active” placebo conditions, providing an appropriate comparator condition. However, due to the relatively low side effect profile of psilocybin compared to ayahuasca—for example lack of nausea and vomiting—it may be more difficult to find an active control condition than demonstrated in the Palhalo-Fontes et al. (10) study. Indeed, studies where an active control is considered critical may have a restricted range of compounds available to them due to the need to provide a convincing control condition.

Other groups have attempted to answer the question of placebo effects, but few with functional connectivity. Of note is the recent study by Sikora et al. (11) [and from the same group (12)] where the effects of “active” placebo—participants had expectations it was a fast-acting antidepressant agent—related to connectivity of the salience network. While this network includes the subgenual cingulate where Carhart-Harris et al. (2) saw effects, and the direction of change was the same, the direct comparisons in the imaging literature are bedeviled by lack of methodological equivalence. Data sharing would not resolve all issues as cohort and scanner effects would still exist, but they would at least allow comparisons to be made with identical processing pipelines.

A shared finding from both Griffith's et al. (1) and Carhart-Harris et al. (2) may in fact be the observed dominant effect of psilocybin over the variance in treatment conditions–highlighted by authors observing non-significance between support conditions (1) and between responders' vs non-responders (2). While converging evidence for both studies is evident, direct experimental data would greatly strengthen claims. As it stands, we cannot yet confidently draw conclusions about added-value of using psilocybin over more conventional treatments, or indeed be specific about psilocybin's mechanisms of action.

In addition to methodological challenges, bloating of public perception toward new and exciting treatment can often be a double-edged sword. Attention may encourage interest and funding but may also raise expectations for clear-cut and quick solutions. Other fields have found lack of control data has led to criticism when comparing current evidence with public expectation [cf: (13, 14)] where authors acknowledge that the “hype” of treatment potential has meant that “misinformation and propagation of poor research methodology can potentially lead to people being harmed, cheated, disappointed, and/or disaffected” (13). This is counter-productive for the popular perception of a treatment and ultimately fatigue interest as results fall short of expectation. Given the potential for novel therapeutic intervention with psilocybin, and indeed other substances in the same field, it is vital the next phase of study centers on rigorous control conditions.

In summary, we propose that progression down two main lines will help take the field from clinically promising to clinically robust. First, the use of controls. We acknowledge that this poses an ethical (withholding treatment) and practical (self-unblinding) problem, however offering psilocybin treatment after the trial and using active comparators (e.g., ketamine) in multi-arm studies may get around these issues, respectively. The ayahuasca literature demonstrates the feasibility of well-designed bespoke comparator conditions. Dose-response studies have a role here as well. Second, the use of additional measures. Through the use of active controls, it would be useful to understand to what extent psilocybin alone may be *specific* to the neurobiological changes observed in Carhart-Harris et al.'s (2) study, and where the limitations and contrasting neural evidence [cf: (11)] may highlight individual and group differences in the usefulness of this treatment.

Psychedelic science now continues to grow and progress forward–with recent studies representing the state of the art. With the rigor of placebo, active control, and mechanistic evidence, we hope that the field of clinical psychedelic science will remain fertile in both public and scientific domains, encouraging continued interest and support from professional bodies.

## Author contributions

JB: initially drafted the manuscript; JB and MM: critically revised the manuscript.

### Conflict of interest statement

The authors declare that the research was conducted in the absence of any commercial or financial relationships that could be construed as a potential conflict of interest.
